# Supplementary material for: Aseismic slip and recent ruptures of persistent asperities along the Alaska-Aleutian subduction zone
Source: Nat Commun. 2022 Jun 2;13:3098. doi: 10.1038/s41467-022-30883-7 (PMC9163073; doi:10.1038/s41467-022-30883-7)
Supplement: Supplementary file 2 — Supplementary Information [file 41467_2022_30883_MOESM2_ESM.pdf]

Supplementary Materials for

Aseismic slip and recent ruptures of persistent asperities along the Alaska-Aleutian subduction zone

Bin Zhao[1,2]*, Roland Bürgmann[3]*, Dongzhen Wang[3], Jian Zhang[1]†, Jiansheng Yu[1], Qi Li[1]

*Corresponding author. Email: zhaobin@cgps.ac.cn (B.Z.) and burgmann@seismo.berkeley.edu (R.B.)

This PDF file includes:

Supplementary Figs. 1 to 23

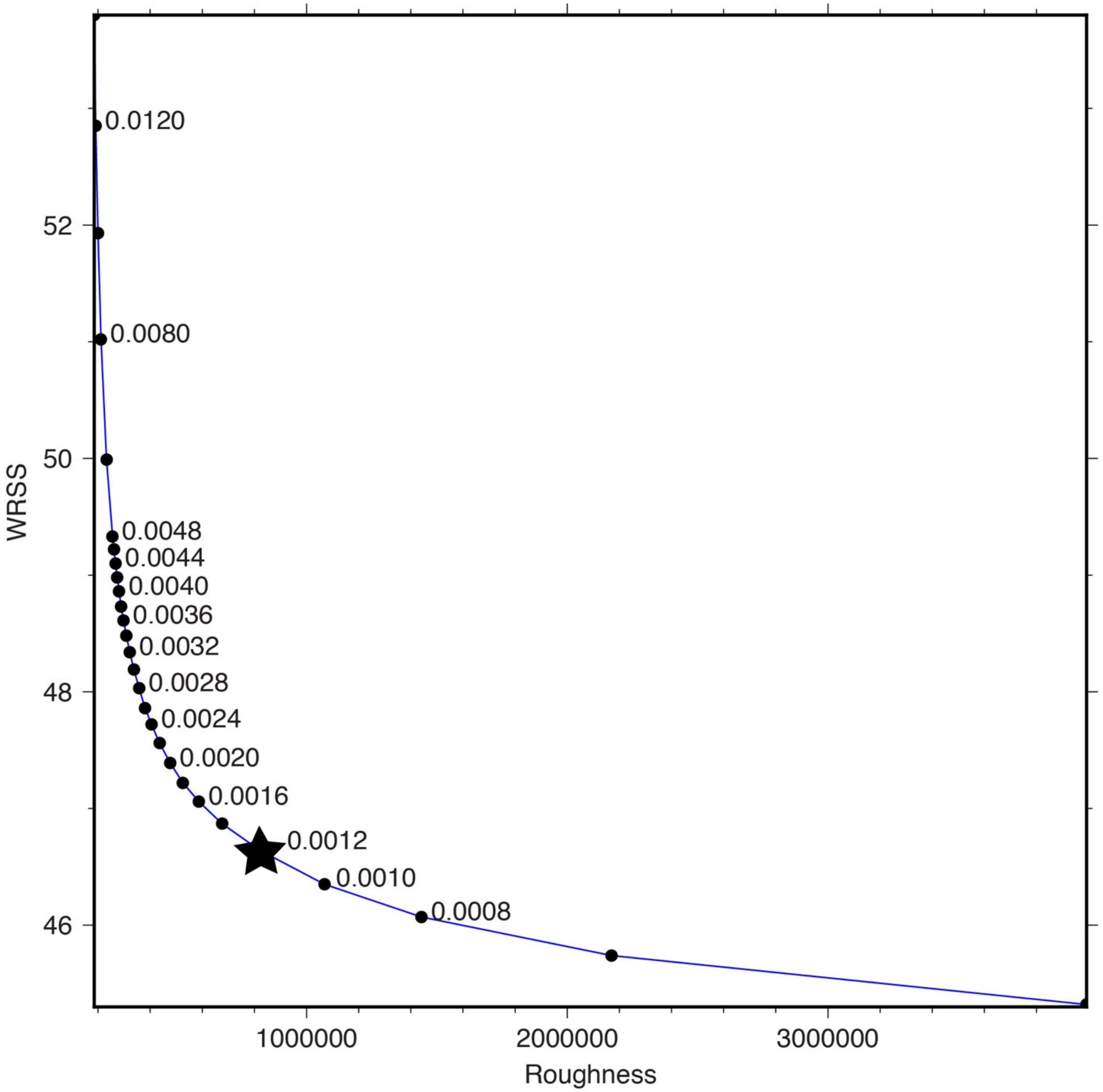

Supplementary Fig. 1. Determination of smoothing factor (β) by visual inspection of the tradeoff curve between model roughness and misfit (Weighted Residual Sum of Squares, WRSS) of the distributed coseismic slip model. The black star is the preferred smoothing factor (0.0012) used in the coseismic slip inversion.

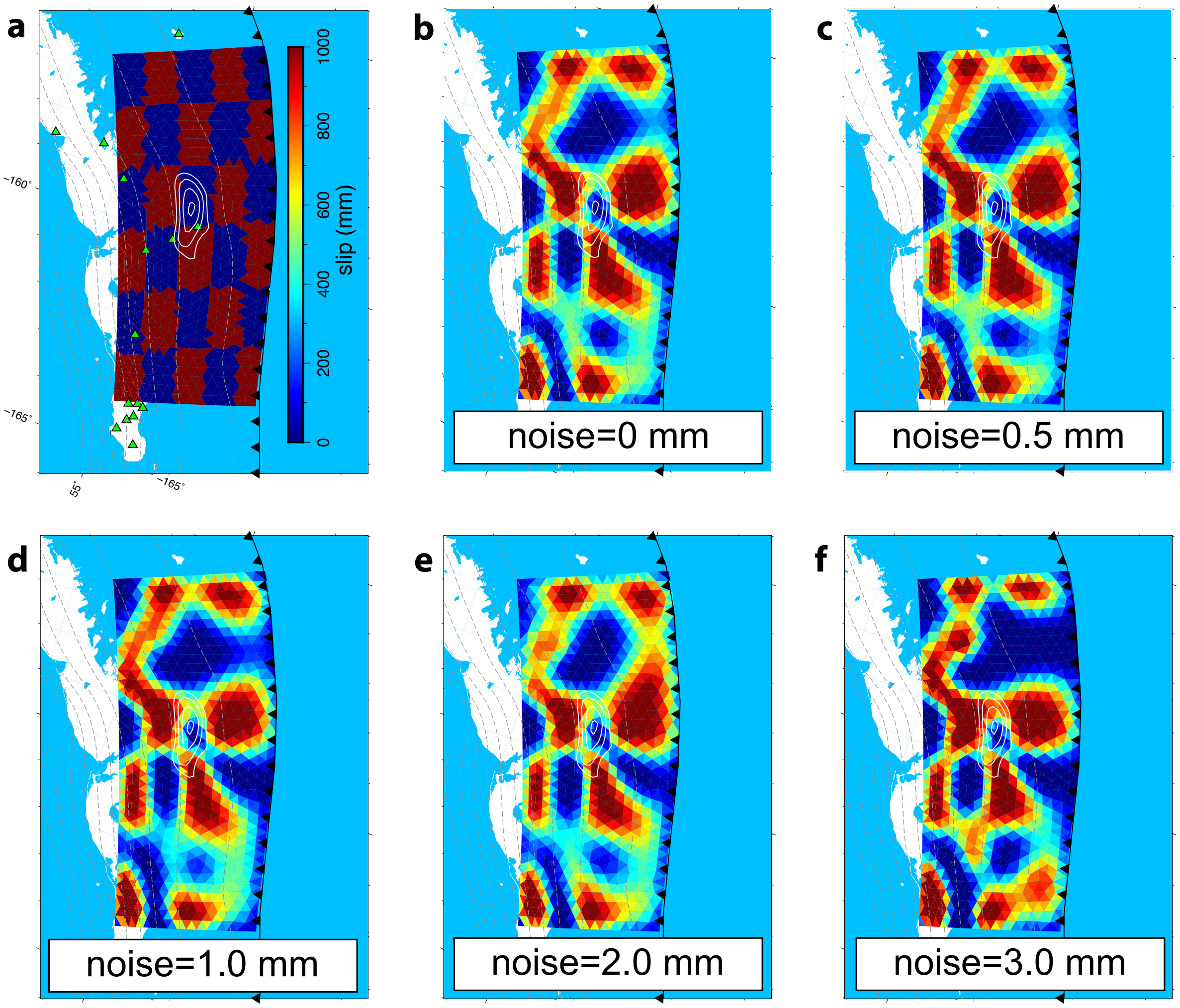

Supplementary Fig. 2. Spatial resolution tests of GPS network distribution. **a** The input slip model. Minimum and maximum slip values are set to 0 (blue) and 1 m (red), respectively. Green triangles are GPS stations. **b-f** Fault slip distribution inverted from synthetic displacements calculated using the input slip model from **a**. We add randomly distributed noise of 0 mm (i.e., no perturbation) (**b**), 0.5 mm (**c**), 1.0 mm (**d**), 2.0 mm (**e**), and 3.0 mm (**f**) to get synthetic displacements before inversion. White lines represent the contours of the coseismic slip distribution of the 2020 Simeonof earthquake shown in Fig. 2.

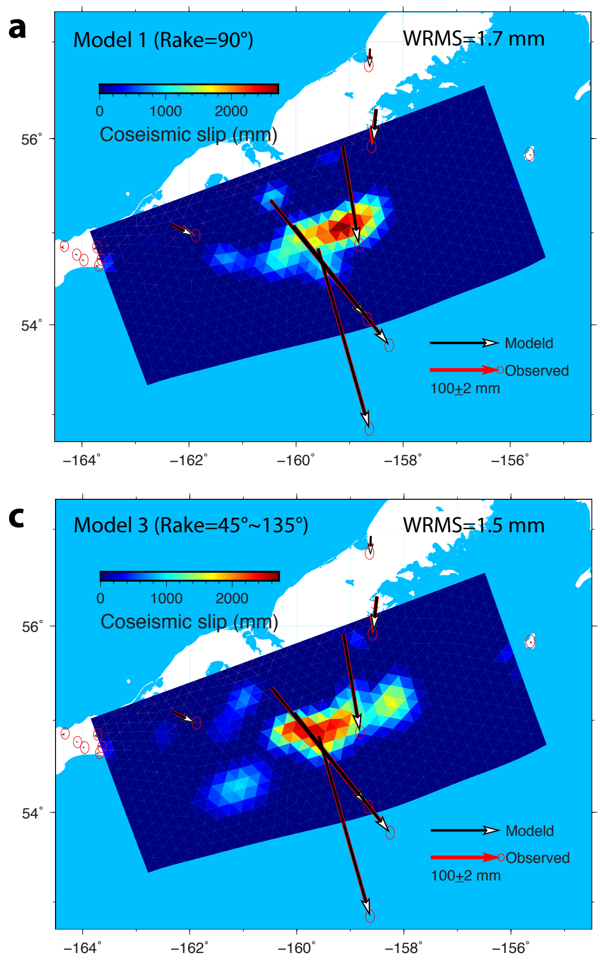

Supplementary Fig. 3. Comparison of coseismic slip models with different rake constraint and regularization methods. The regularization method of Maerten et al.[62] is used in **a** and **c**, and the zeroth-order Tikhonov approach is used in **b** and **d**. The WRMS misfit for each model is labelled in each panel.

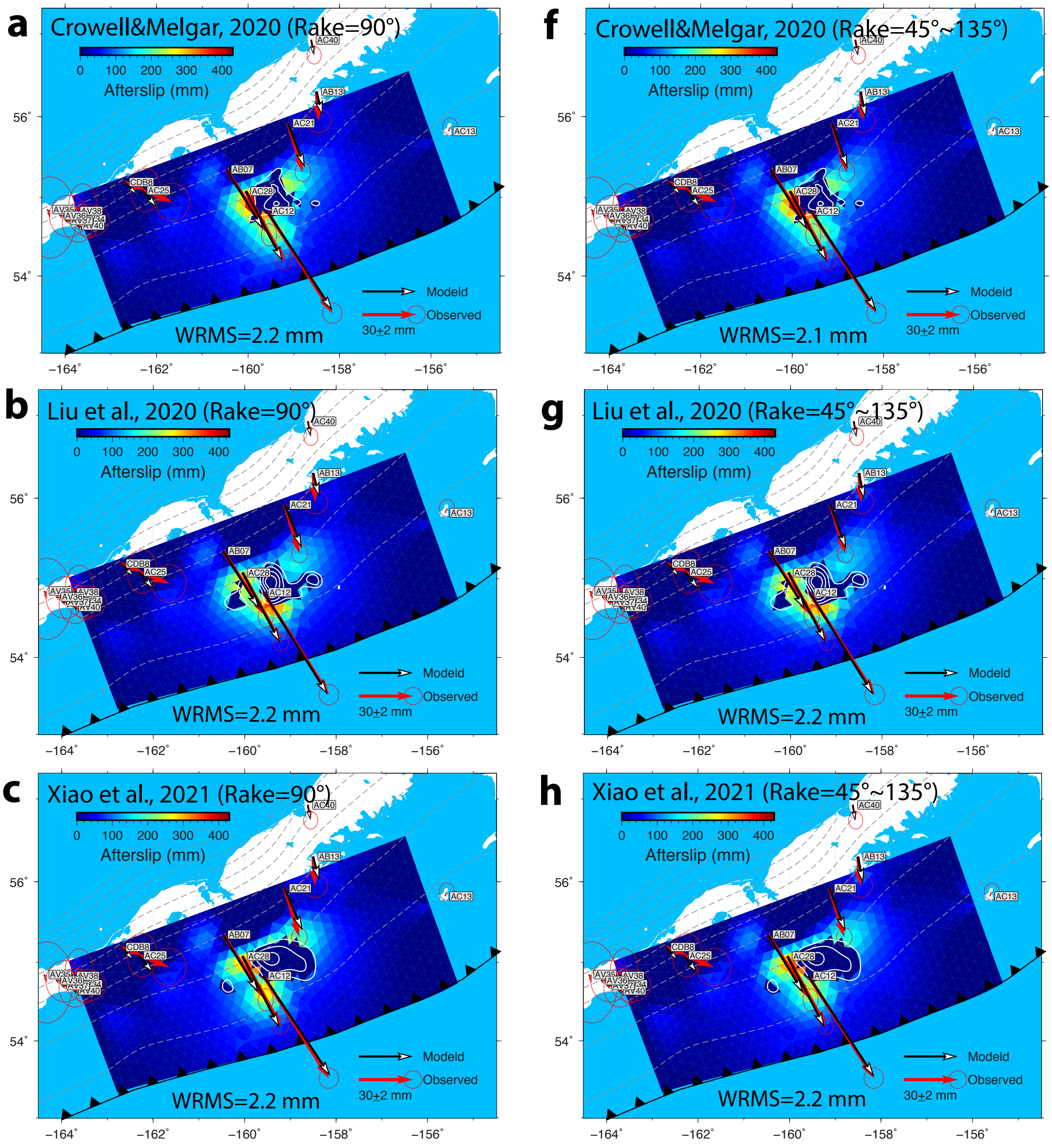
a
Crowell&Melgar, 2020 (Rake=90°)
Afterslip (mm)
WRMS=2.2 mm
f
Crowell&Melgar, 2020 (Rake=45°~135°)
Afterslip (mm)
WRMS=2.1 mm
b
Liu et al., 2020 (Rake=90°)
Afterslip (mm)
WRMS=2.2 mm
g
Liu et al., 2020 (Rake=45°~135°)
Afterslip (mm)
WRMS=2.2 mm
c
Xiao et al., 2021 (Rake=90°)
Afterslip (mm)
WRMS=2.2 mm
h
Xiao et al., 2021 (Rake=45°~135°)
Afterslip (mm)
WRMS=2.2 mm
Modeld
Observed
30±2 mm

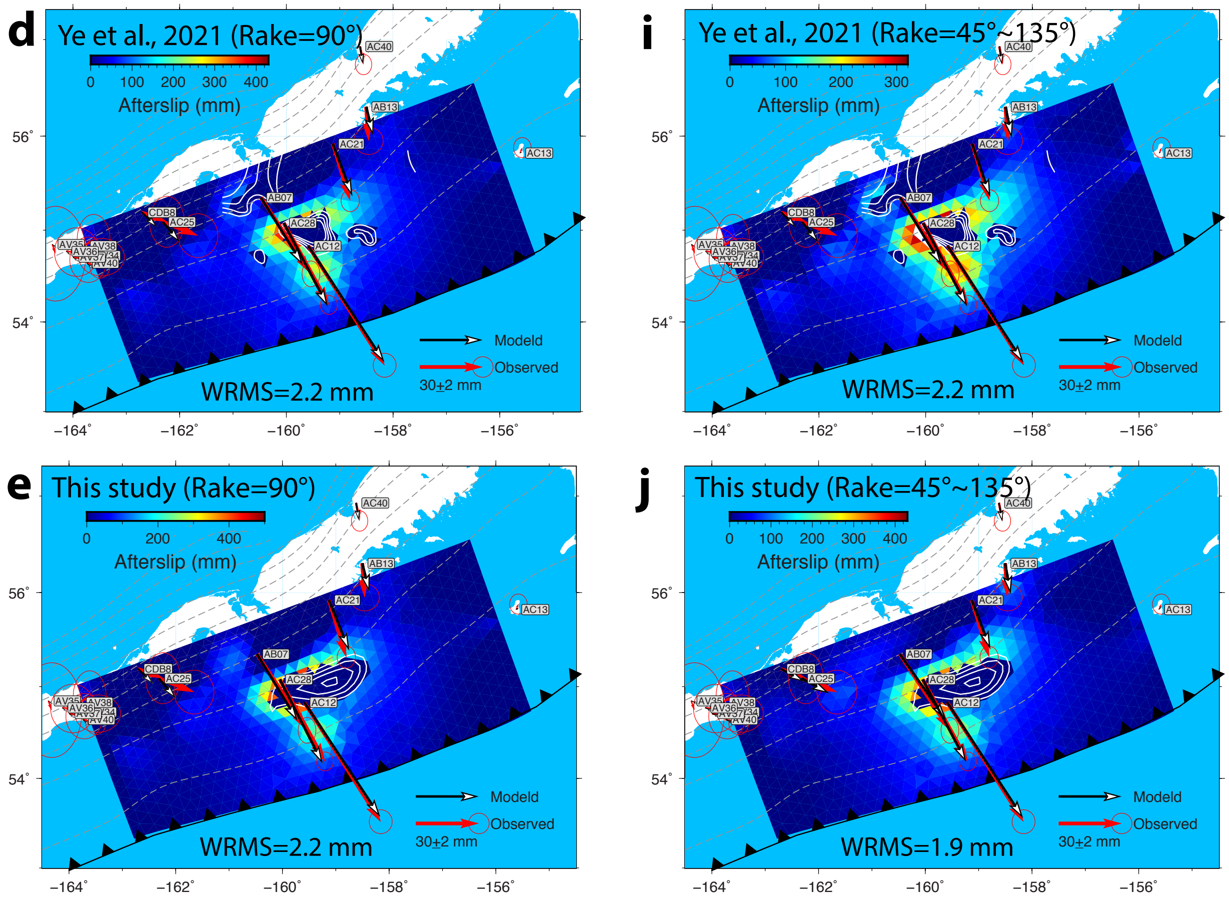

Supplementary Fig. 4. Kinematic afterslip models with the zero-slip constraint based on different coseismic slip models with different rake constraints. Left panels (**a-e**) show coseismic models with rake fixed at 90°, and right panels (**f-j**) are models with rake constraint between 45°~135°. The WRMS misfit for each model is labelled in each panel.

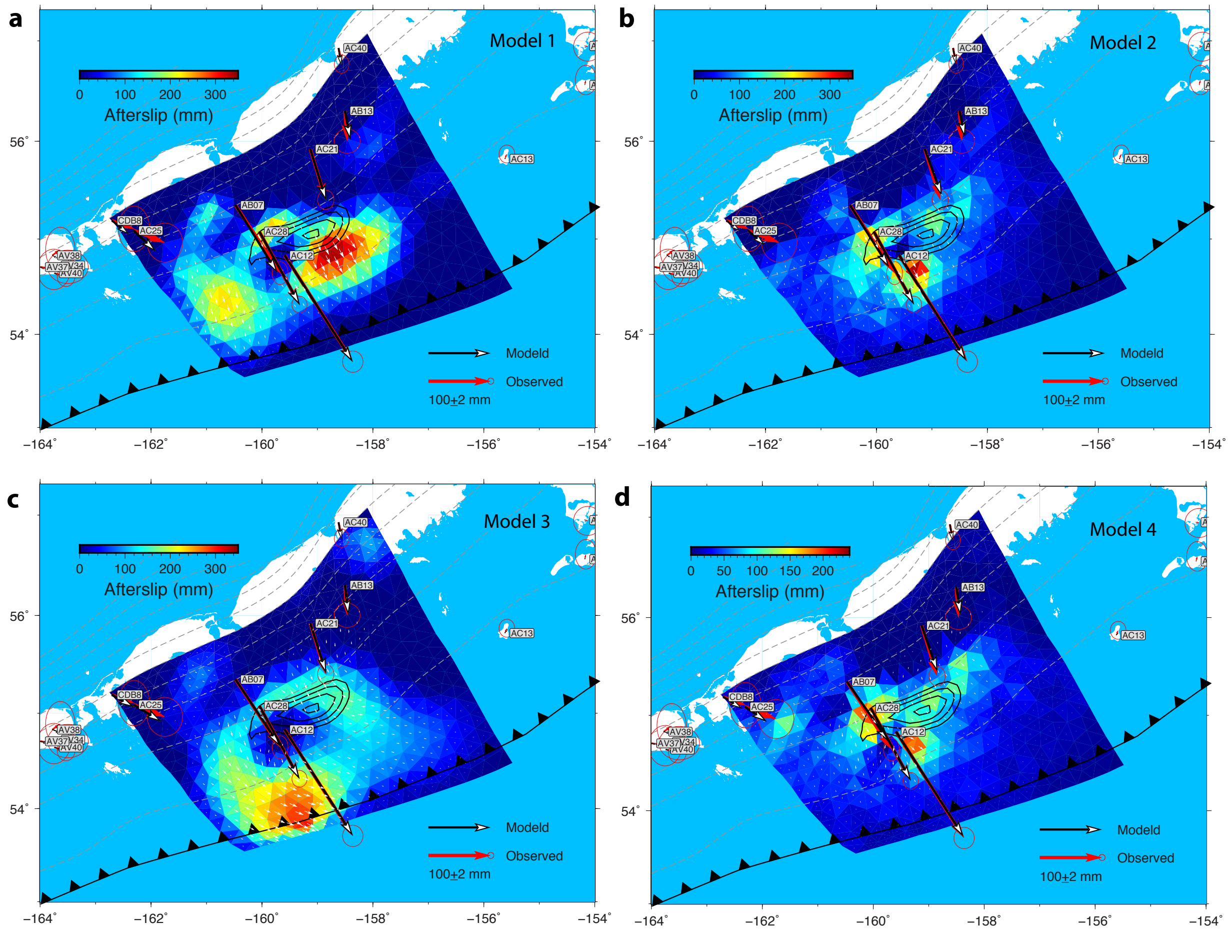

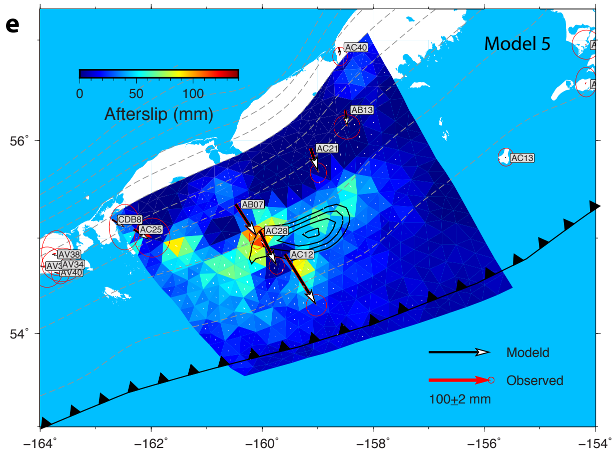

f

A summary of different kinematic afterslip models

Model name	Rake constraint (deg)	Regularization	Time interval (days)
Model 1	90	Maerten05	87
Model 2	90	Tikhonov	87
Model 3	[35, 135]	Maerten05	87
Model 4	[35, 135]	Tikhonov	87
Model 5	[35, 135]	Tikhonov	10

Supplementary Fig. 5. Kinematic afterslip models with different rake constraints and regularization methods and time intervals, but without zero-slip constraint. **a–e** Kinematic afterslip distributions with model parameterizations of models summarized in **f**. The triangular mesh is the same as that used in Crowell & Melgar[27].

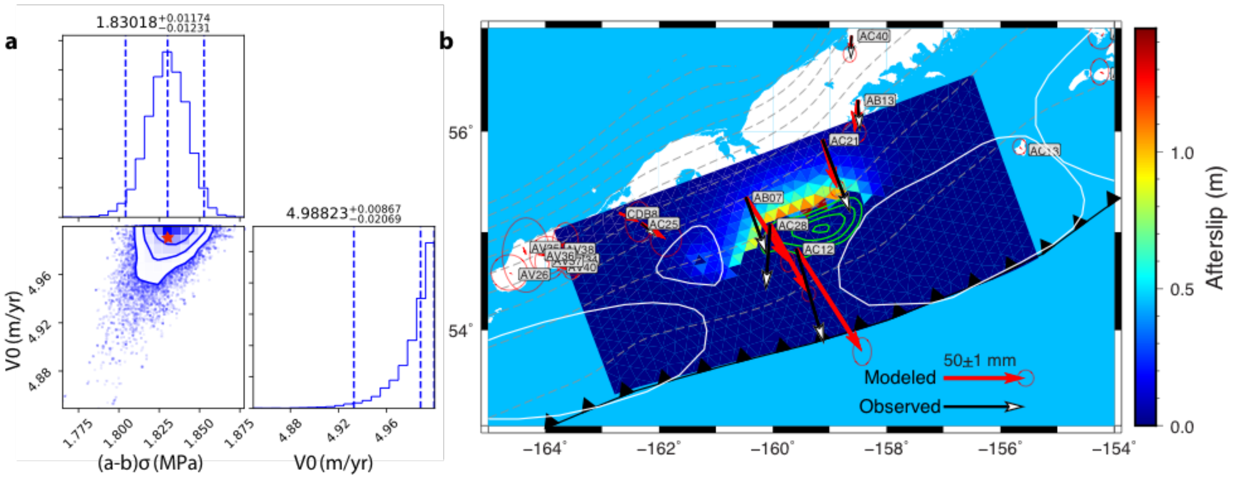

Supplementary Fig. 6. Results of frictional afterslip model for the first scenario. In this case, afterslip on the slab interface is only permitted on the downdip side of the modelled coseismic rupture. **a** Marginal posteriors of frictional parameter $(\mathrm{a}-\mathrm{b})\sigma_n$ and reference velocity v_0. The blue lines in the histograms denote 95% confidence level error bounds on each parameter. Red star represents the best-fit constitutive parameter values. **b** Distribution of stress-driven afterslip in the first 87-day interval and comparison between the GPS observed postseismic displacements with the predictions. Dashed grey lines depict the depth contours from the Slab2 model, white curves are rupture zones of historical earthquakes, and green contours represent the coseismic slip model of the 2020 Mw 7.8 Simeonof earthquake determined in this study.

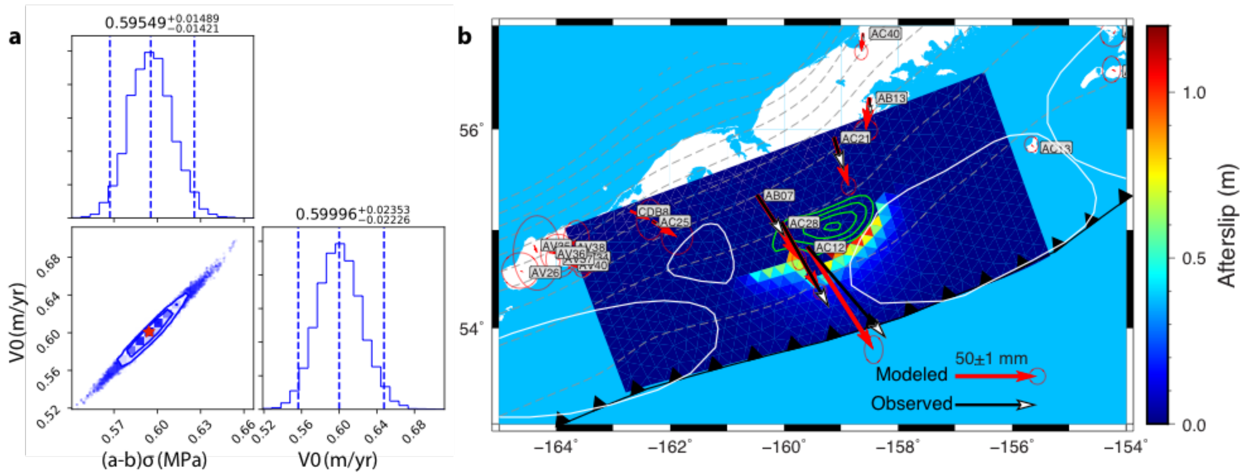

Supplementary Fig. 7. The same as Supplementary Fig. 6 but for the second scenario. In this case, stress-driven afterslip is only allowed on the updip extension of the coseismic slip zone.

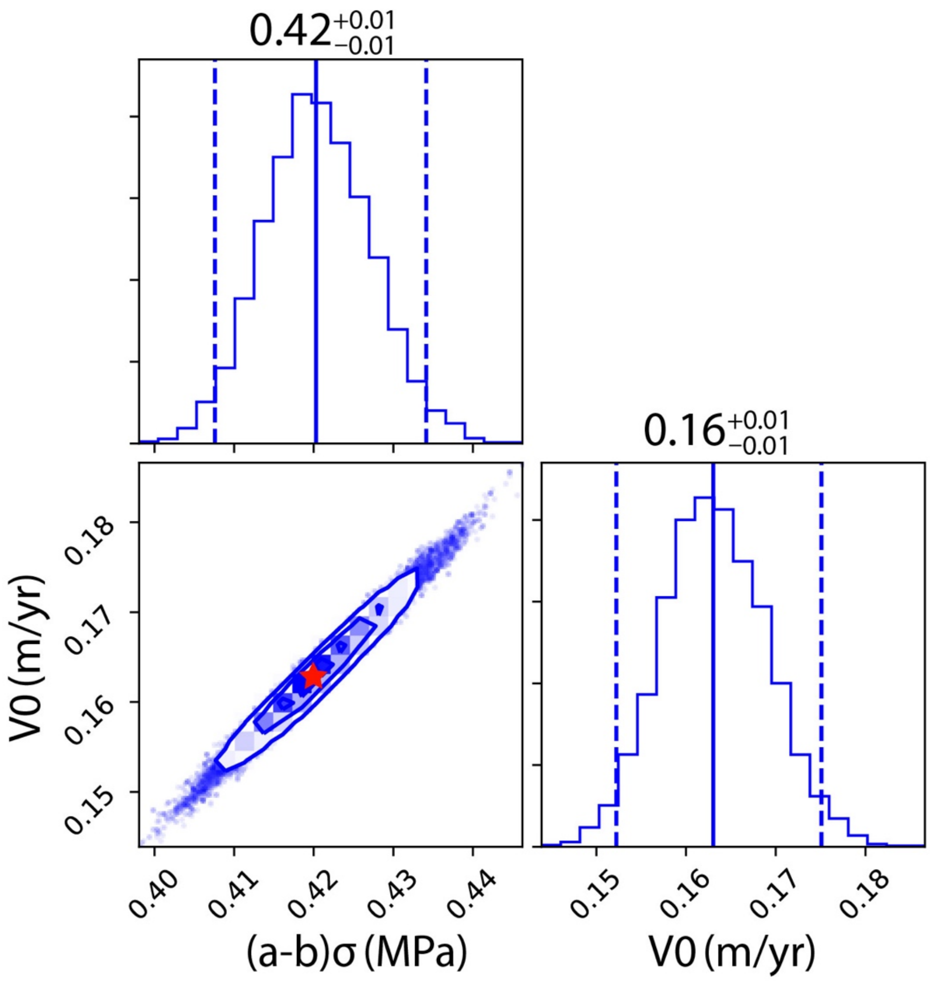

Supplementary Fig. 8. Determination of frictional properties for the third stress-driven afterslip scenario (afterslip fully surrounding the rupture). In this case, we assume that the frictional properties are uniform on the plate interface away from the coseismic slip zone.

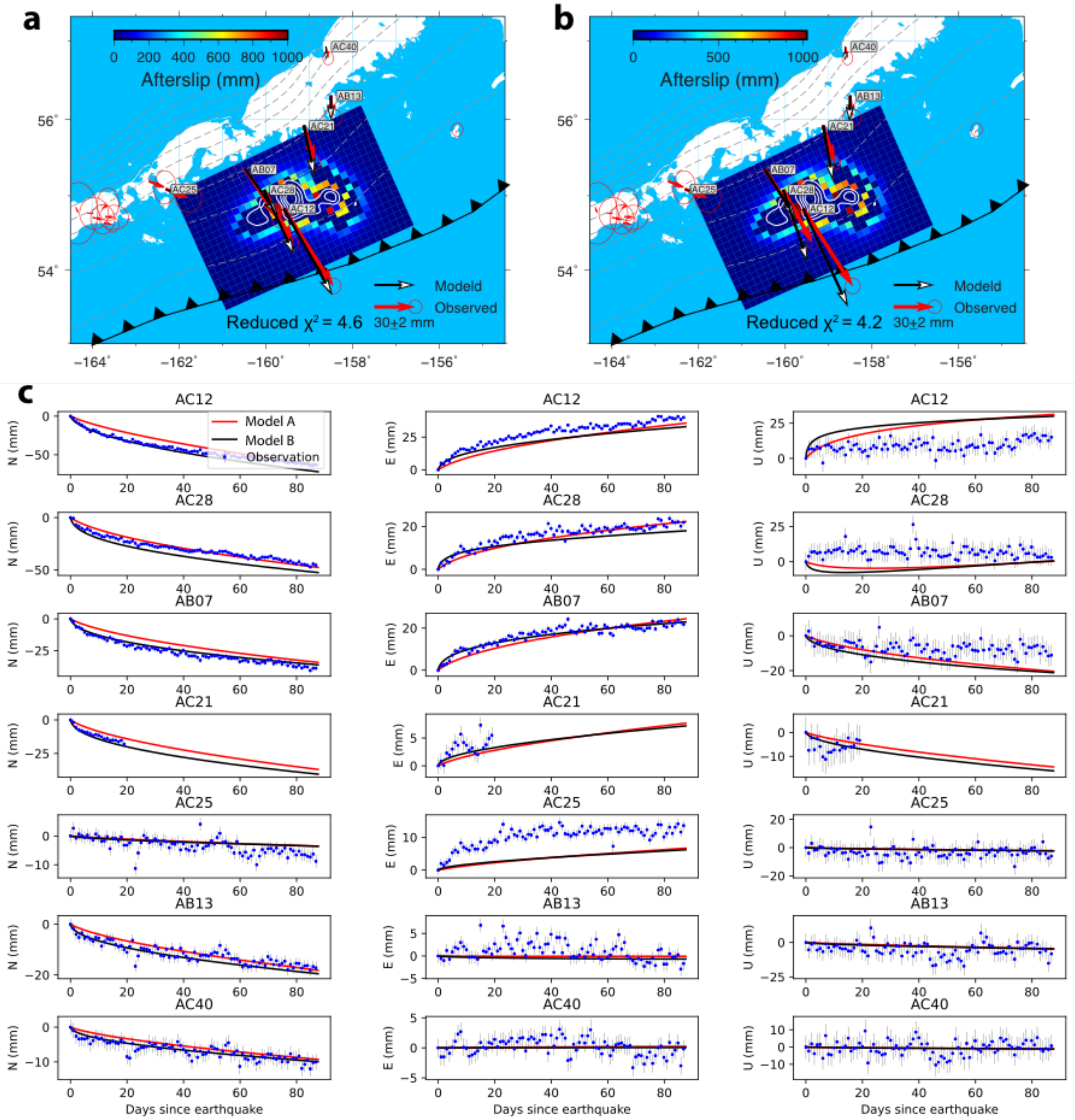

Supplementary Fig. 9. Stress-driven afterslip model based on coseismic slip model from Liu et al.[28]. **a** and **b** Show the cumulative 87-day afterslip distributions with the rake of the stress-driven afterslip fixed at 90° and with variable rake determined according to the shear stress evolution, respectively. **c** Illustrates the comparison between GPS observed and modeled postseismic time series. Models in **a** and **b** yield normalized χ^2 of 4.6 and 4.2 based on equation (4), respectively, both are larger than that of the preferred model (normalized χ^2=3.0) presented in the main text.

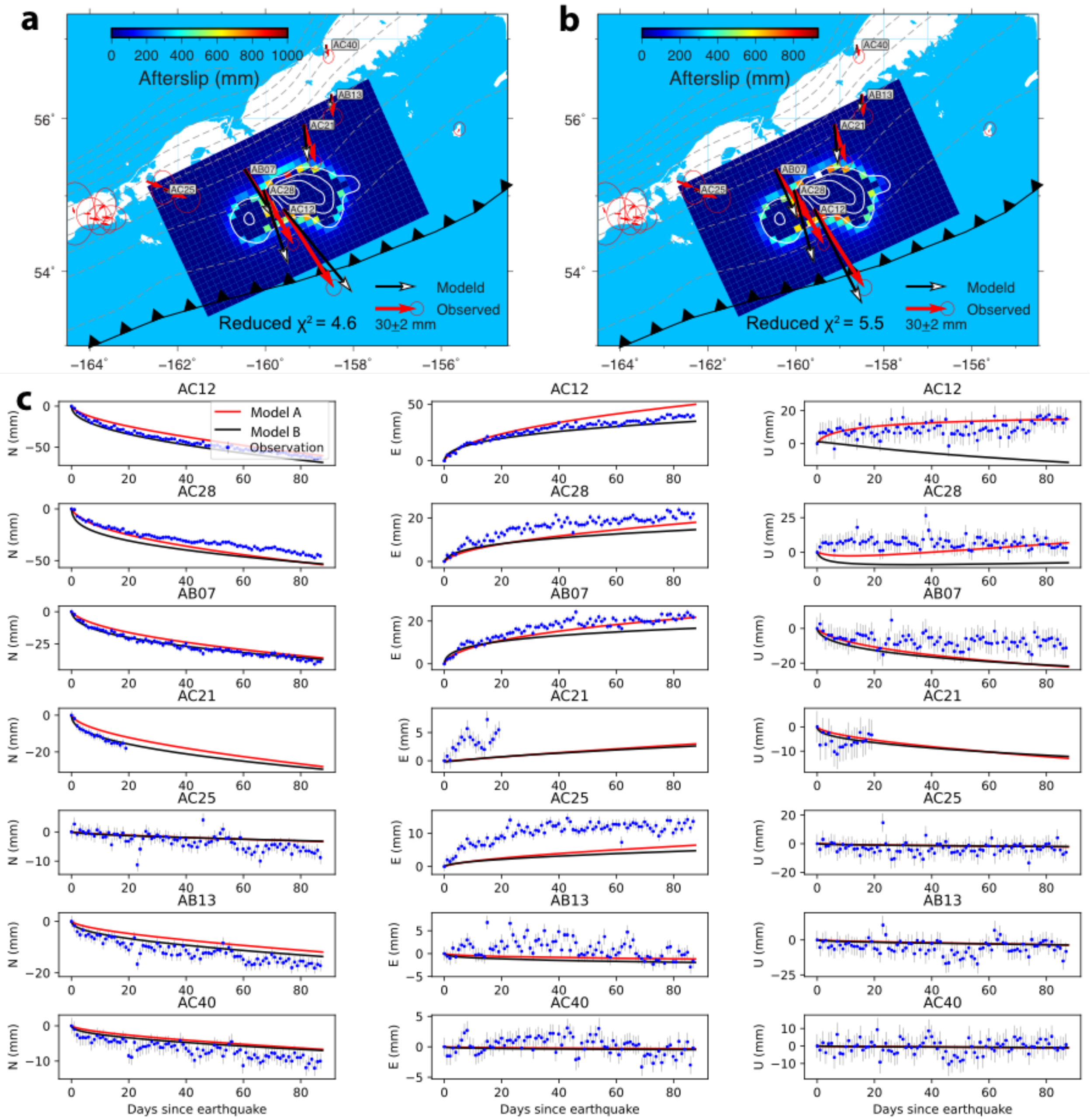

Supplementary Fig. 10. The same as Supplementary Fig. 9 but with coseismic slip model from Xiao et al.[30]. Models in **a** and **b** yield normalized χ^2 of 4.6 and 5.5 based on equation (4), respectively.

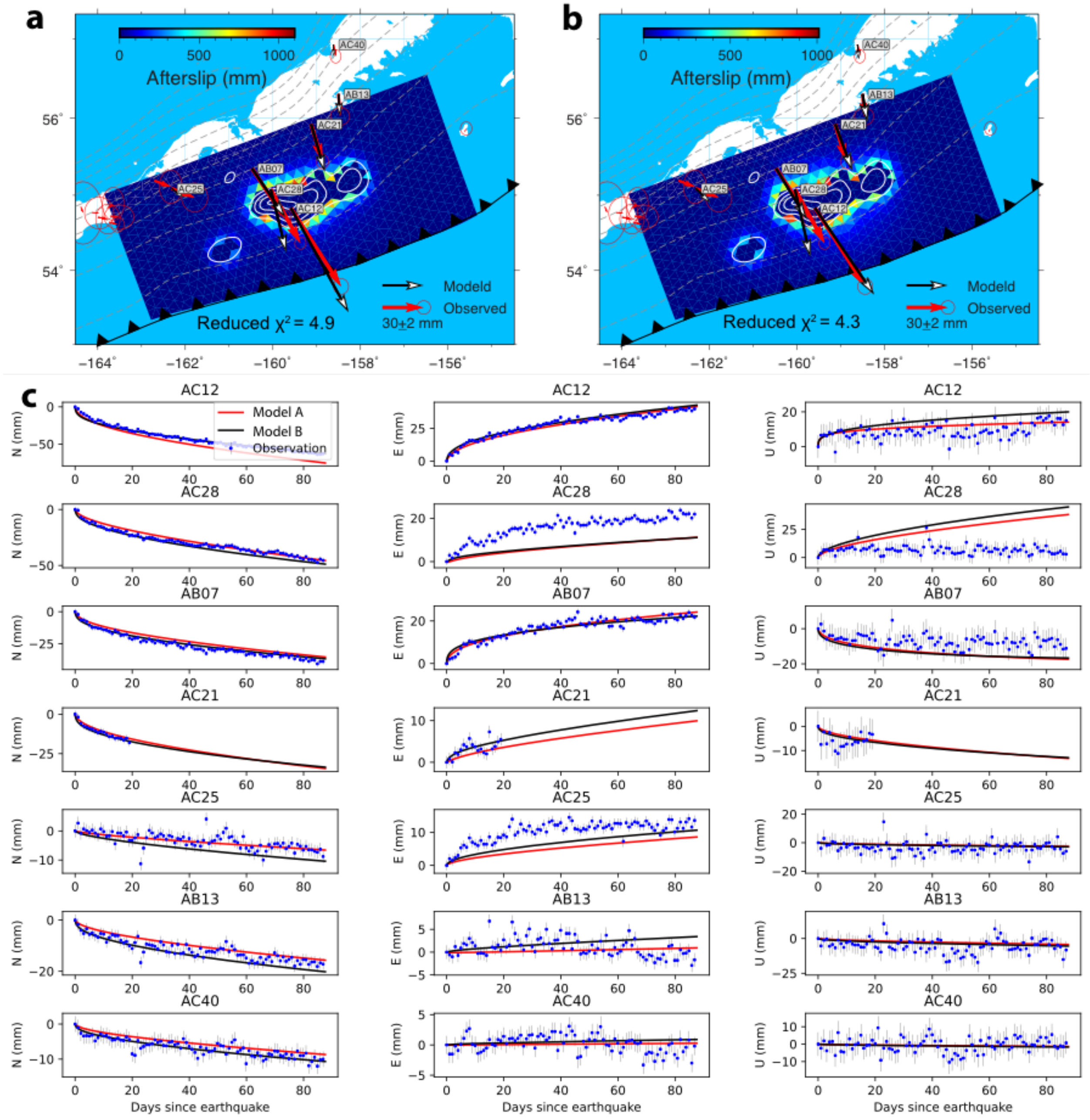

Supplementary Fig. 11. The same as Supplementary Fig. 9 but with the coseismic slip model shown in Supplementary Fig. 3c. Models in **a** and **b** yield normalized χ^2 of 4.9 and 4.3 based on equation (4), respectively.

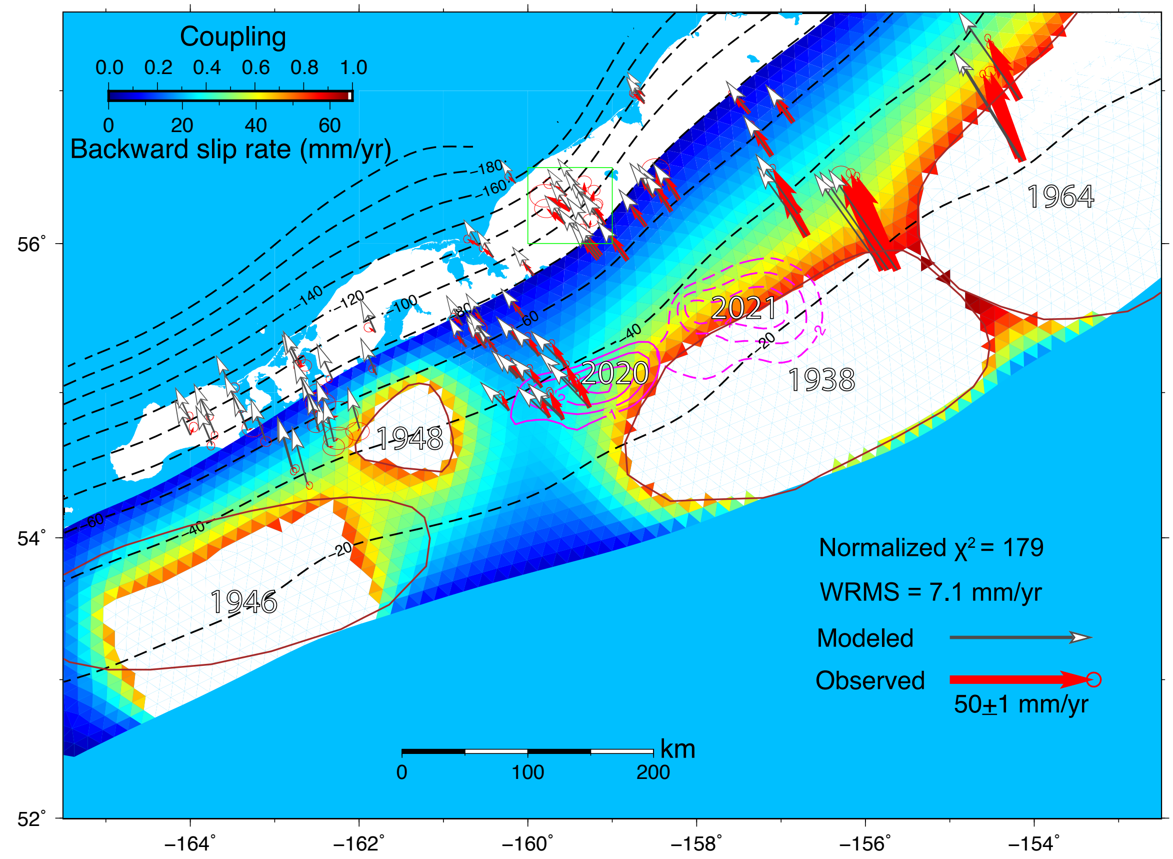

Supplementary Fig. 12. Interseismic backward slip rate (coupling) distribution in the Alaska subduction zone. In this scenario, the distribution of locked asperities is based on the inferred extent of historical earthquakes. Red outlines labeled with year are the rupture zones of large earthquakes inferred from aftershock distributions[64,65]. We approximate the rupture zone of the 1946 Unimak earthquake according to López and Okal[17]. Solid and dashed magenta lines represent the contours of the coseismic slip distribution of the 2020 Simeonof and 2021 Chignik earthquakes, respectively. The modeled velocities with respect to the North American plate greatly overpredict the magnitude of the observed velocities, producing a very large normalized χ^2 of 179 and a WRMS of 7.1 mm/yr. Shown in white are locked asperities with a backward slip rate of 65 mm/yr, the relative plate convergence rate between the Pacific plate and North American plate. The colors of triangular elements denote backward slip rate calculated from the forward boundary element method (BEM) model. Black and red arrows show the observed and predicted station velocities. The GPS-observed interseismic velocities are from Li & Freymueller[22] after removing trench-parallel forearc block motion. Black lines depict the depth contours from the Slab2 model.

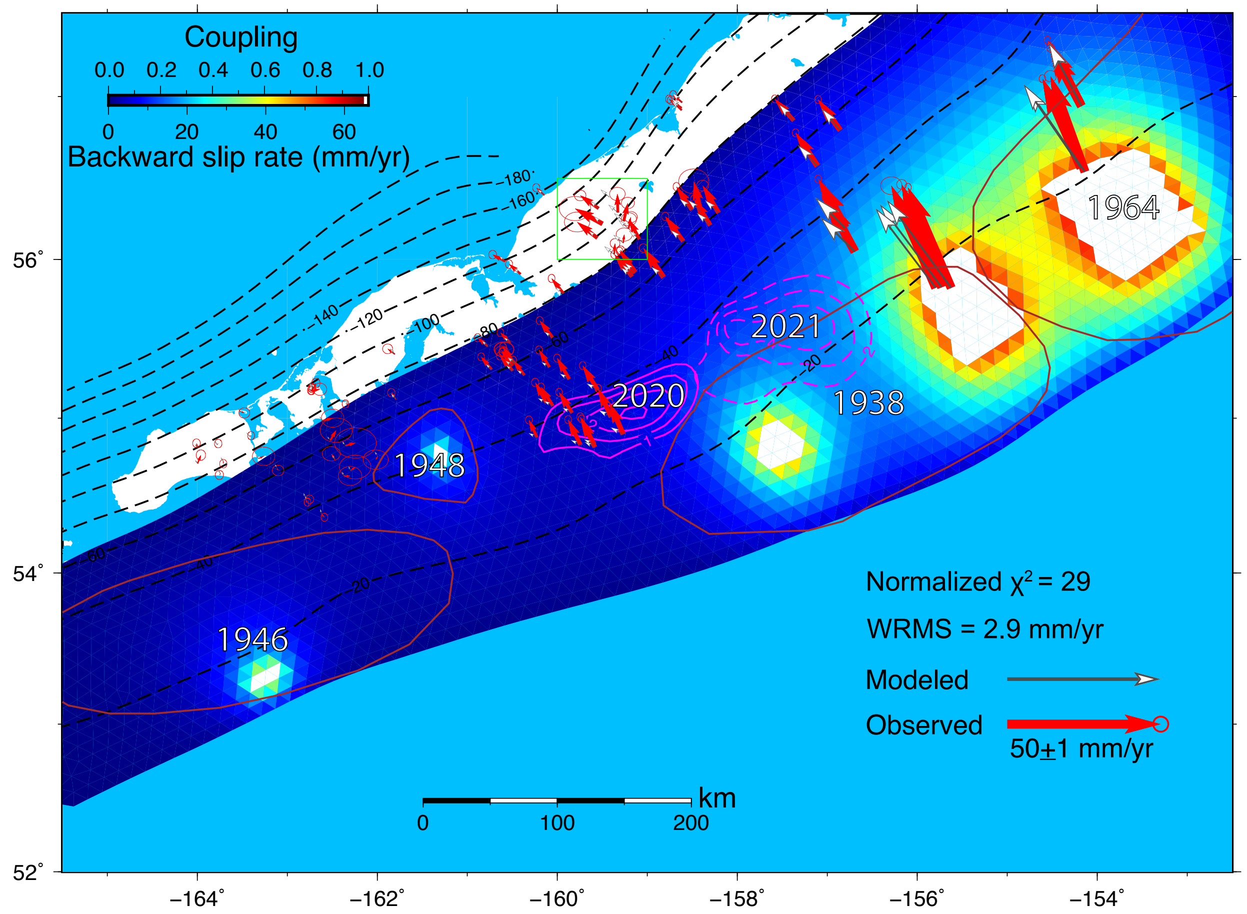

Supplementary Fig. 13. Same as Supplementary Fig. 12, but in this scenario, we show the case in which only the core zones of historical earthquakes are fully locked, yielding a normalized χ^2 of 29 and a WRMS value of 2.9 mm/yr.

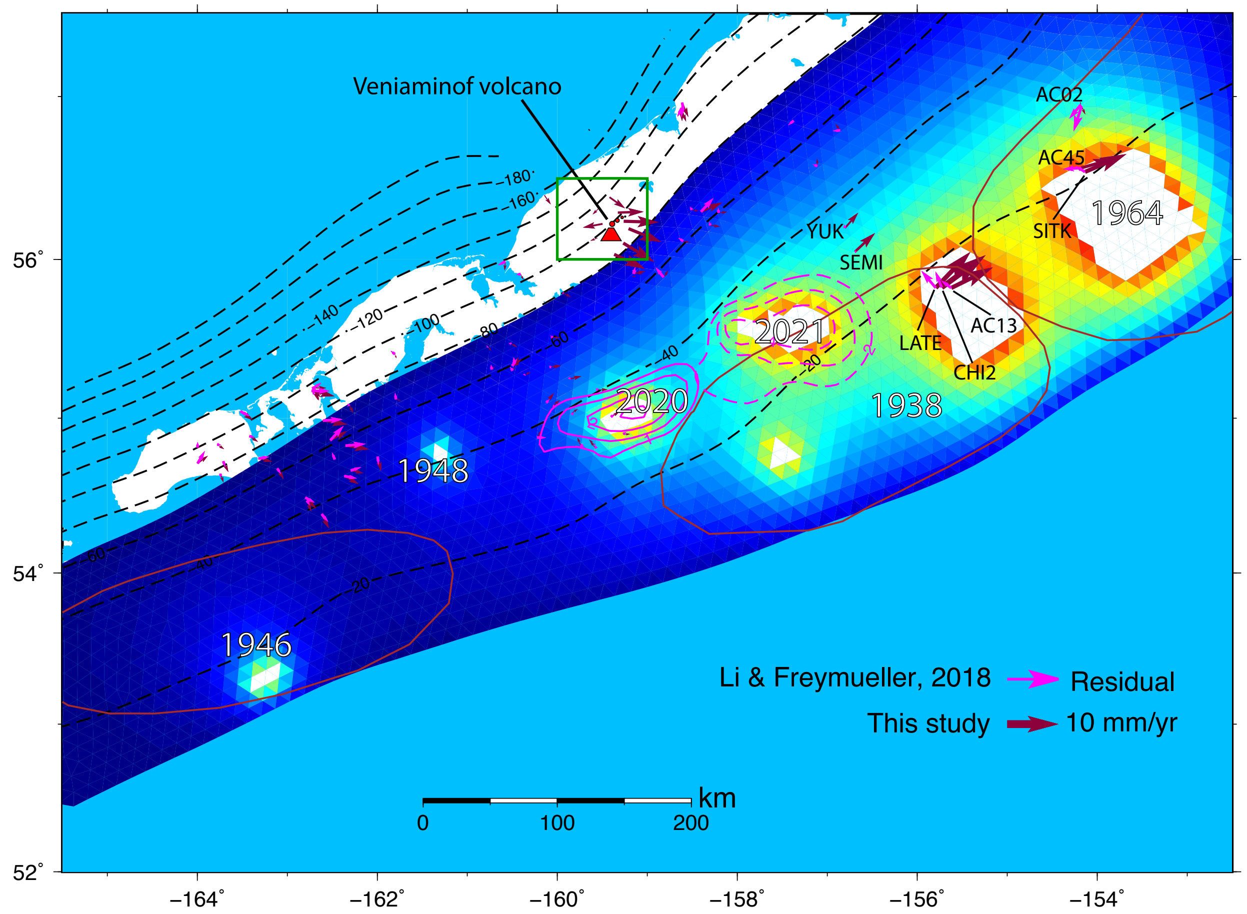

Supplementary Fig. 14. Comparison of residual interseismic velocities between the GPS observed and predicted velocities from different interseismic models. Colors of triangular elements denote backward slip rate (coupling) calculated from the forward BEM model in Fig. 5. Note that sites on and around Mt. Veniaminof volcano in the green rectangular were not used in misfit calculation.

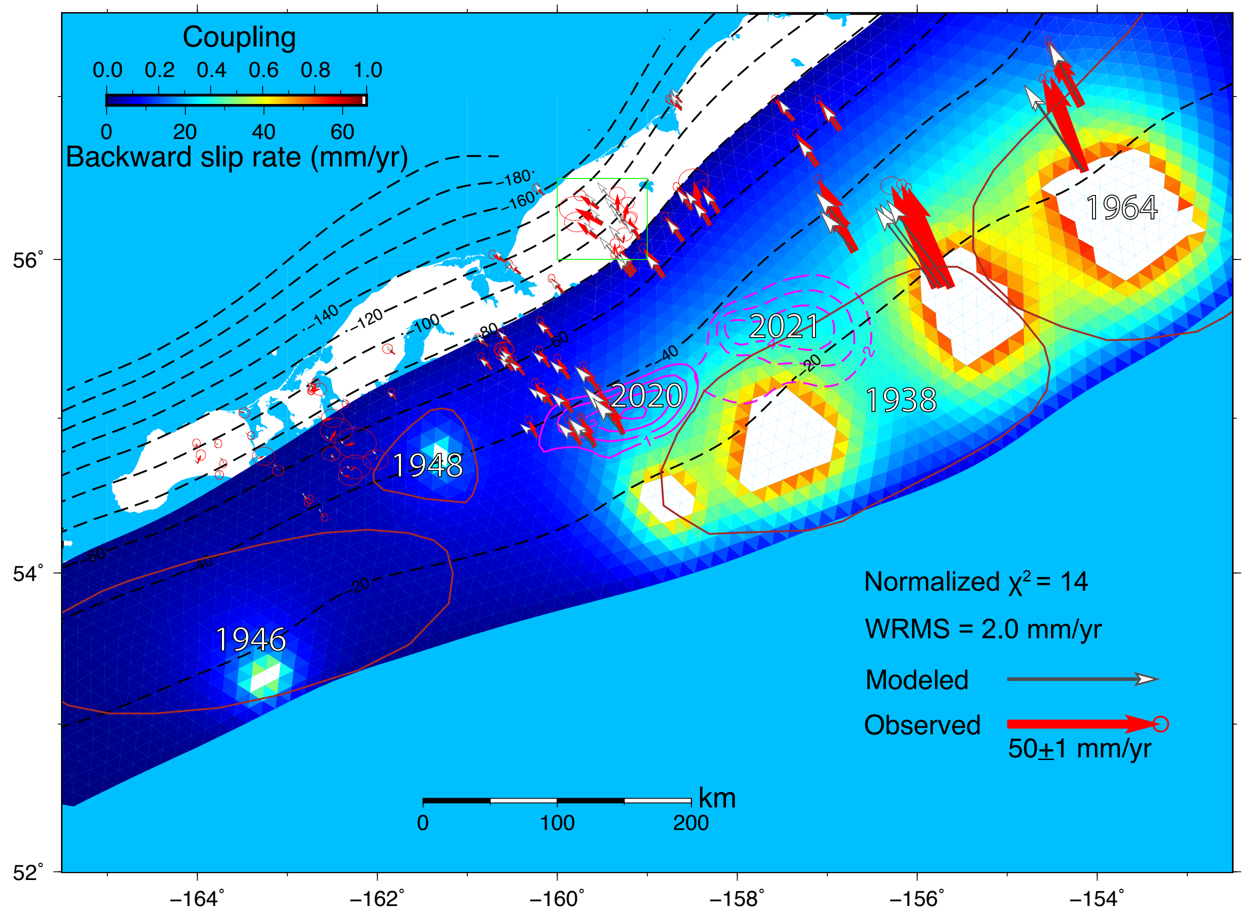

Supplementary Fig. 15. Same as Fig. 5, but in this scenario, we show that somewhat larger asperities along the Shumagin Gap, located updip of the recent earthquakes above 20 km depth, can equally well fit the observed GPS velocities on the Shumagin Islands, yielding a normalized χ^2 of 14 and a WRMS value of 2.0 mm/yr.

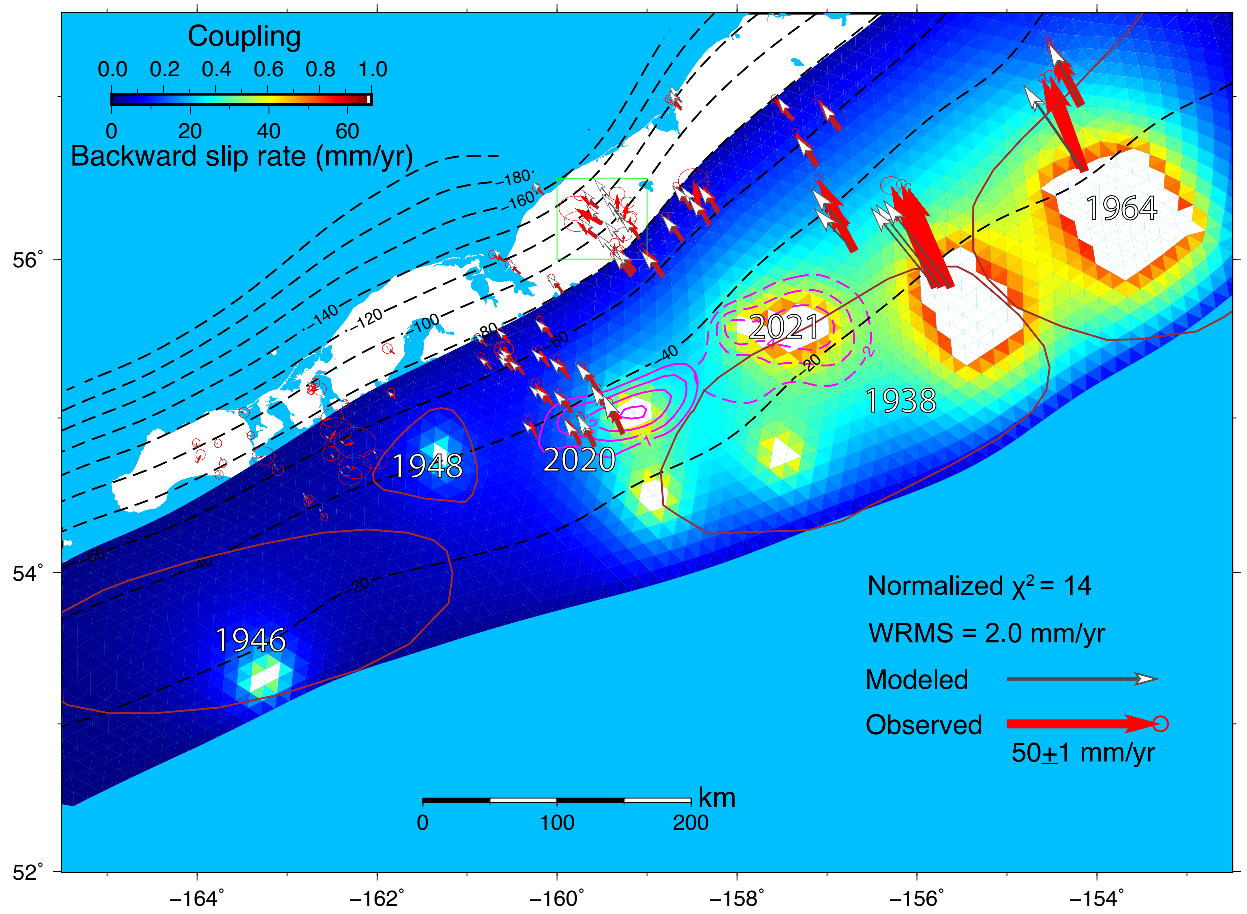

Supplementary Fig. 16. Same as Fig. 5, but in this scenario, we show that a relatively small asperity corresponding to the 2020 earthquake and an additional shallower asperity above 20 km can equally well fit the observed GPS velocities on the Shumagin Islands, yielding a normalized χ^2 of 14 and a WRMS value of 2.0 mm/yr.

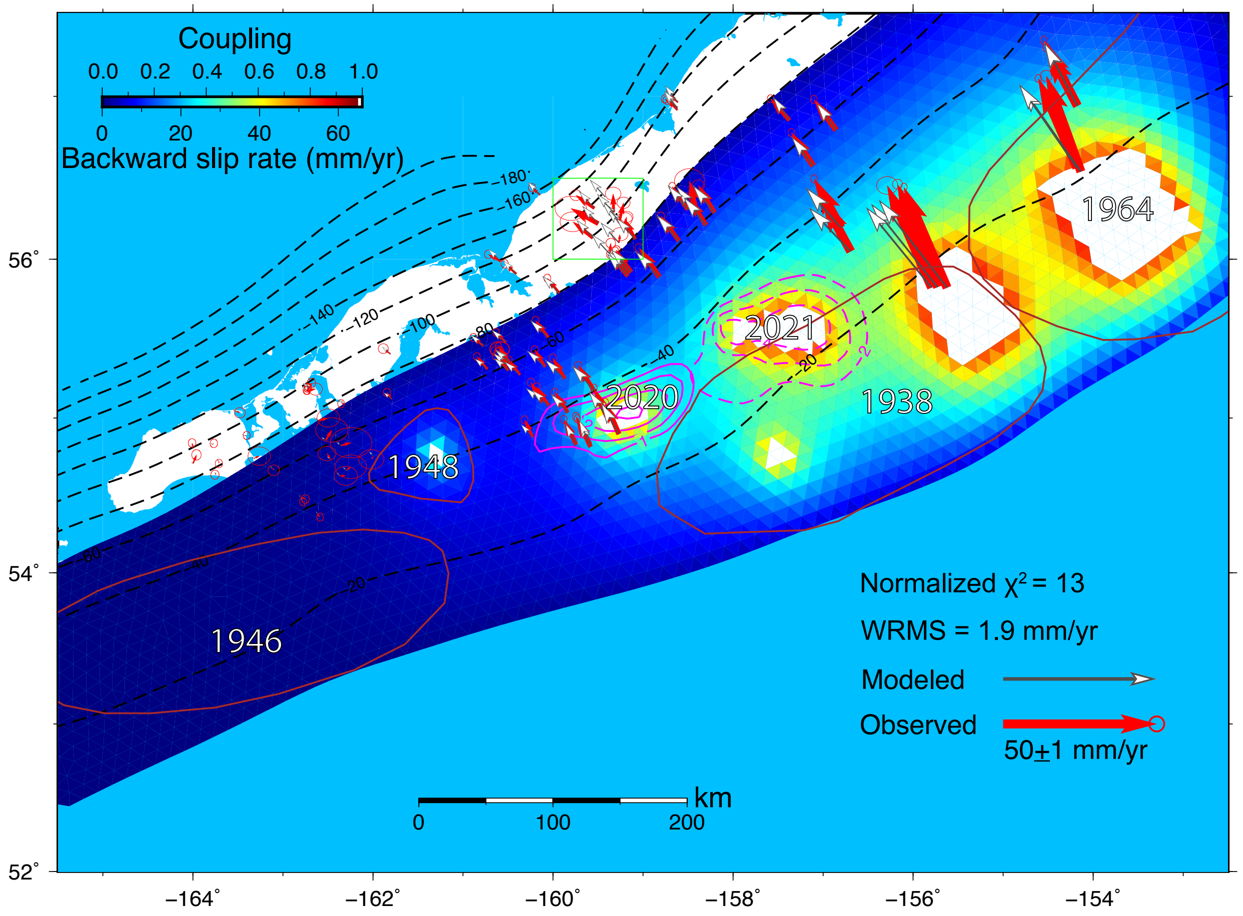

Supplementary Fig. 17. Same as Fig. 5, but in this scenario, we show that the data misfit between the observed and modeled velocities decreases slightly (normalized χ^2=13, WRMS=1.9 mm/yr), if the asperity corresponding to the 1946 tsunami earthquake is excluded. The lack of GPS stations to the W of longitude 164°W severely limits the ability to constrain coupling in the westernmost section of the 1946 aftershock zone.

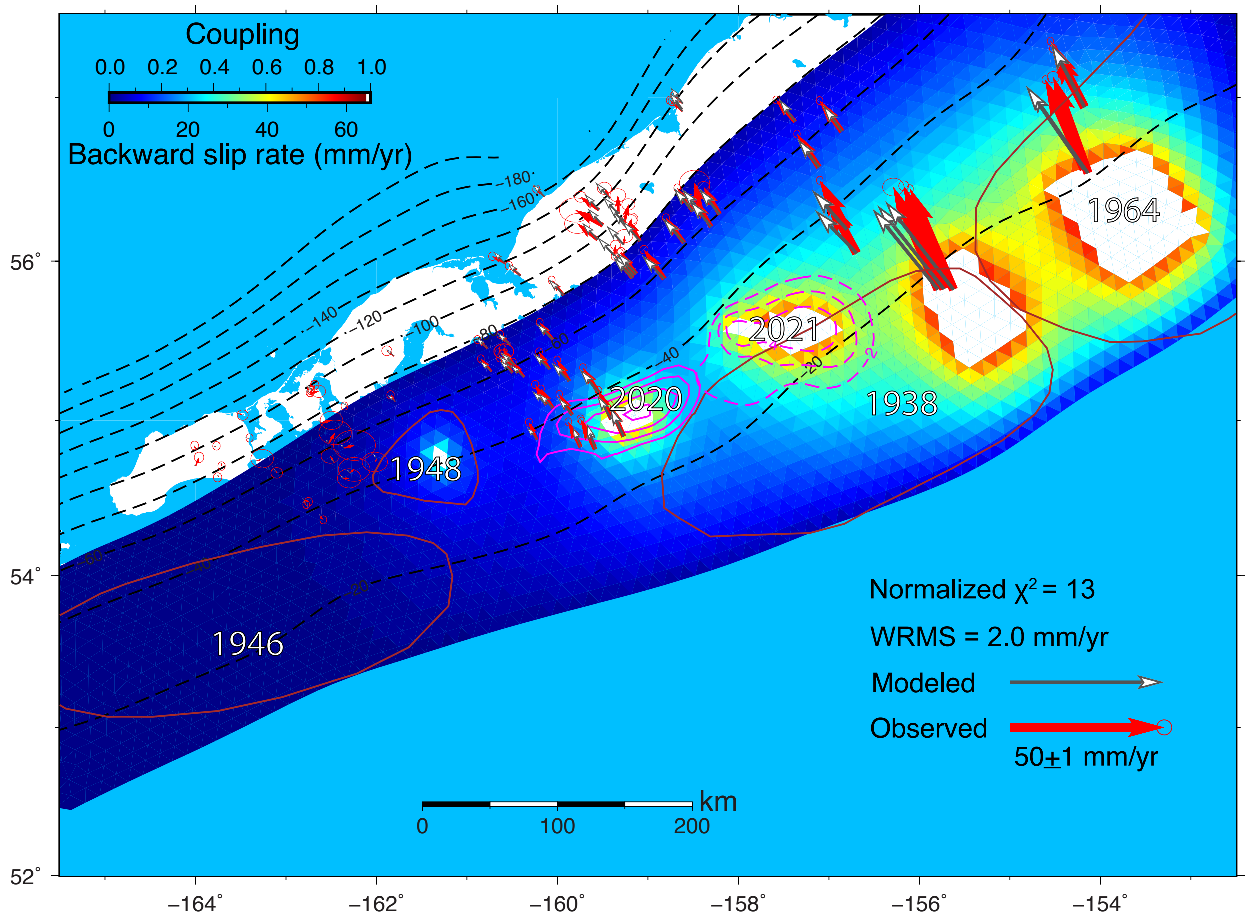

Supplementary Fig. 18. Plausible backslip rate (coupling) distribution with the least complexity. The asperities corresponding to the 1946 tsunami earthquake and to the western rupture of the 1938 earthquake are excluded. This model achieves a fit comparable to that shown in Fig. 5 and Fig. 15-17 (normalized χ^2 = 13, WRMS=2.0 mm/yr).

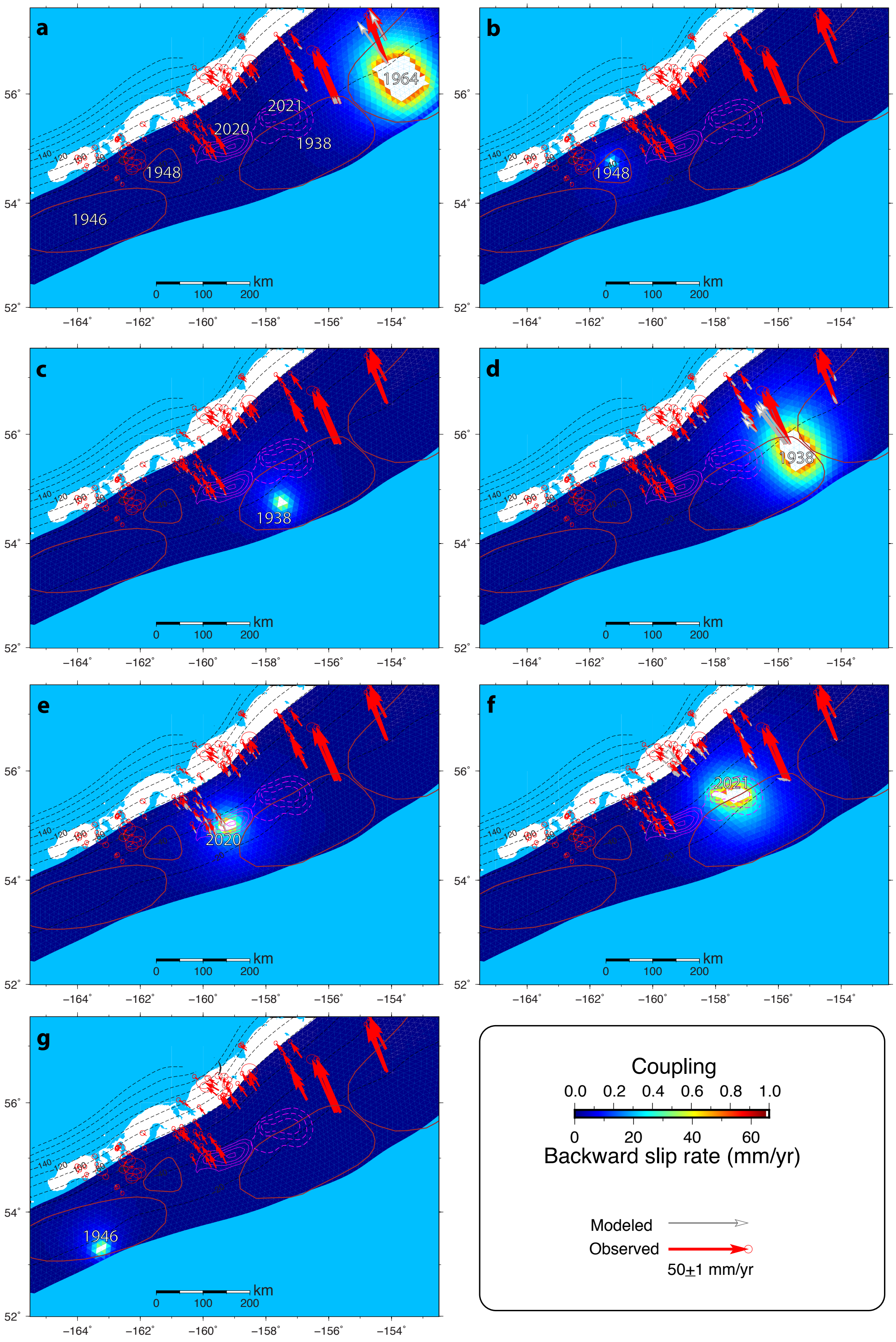

Supplementary Fig. 19. Comparison of GPS observed interseismic velocities to predictions from individual locked asperities calculated from the forward BEM models.

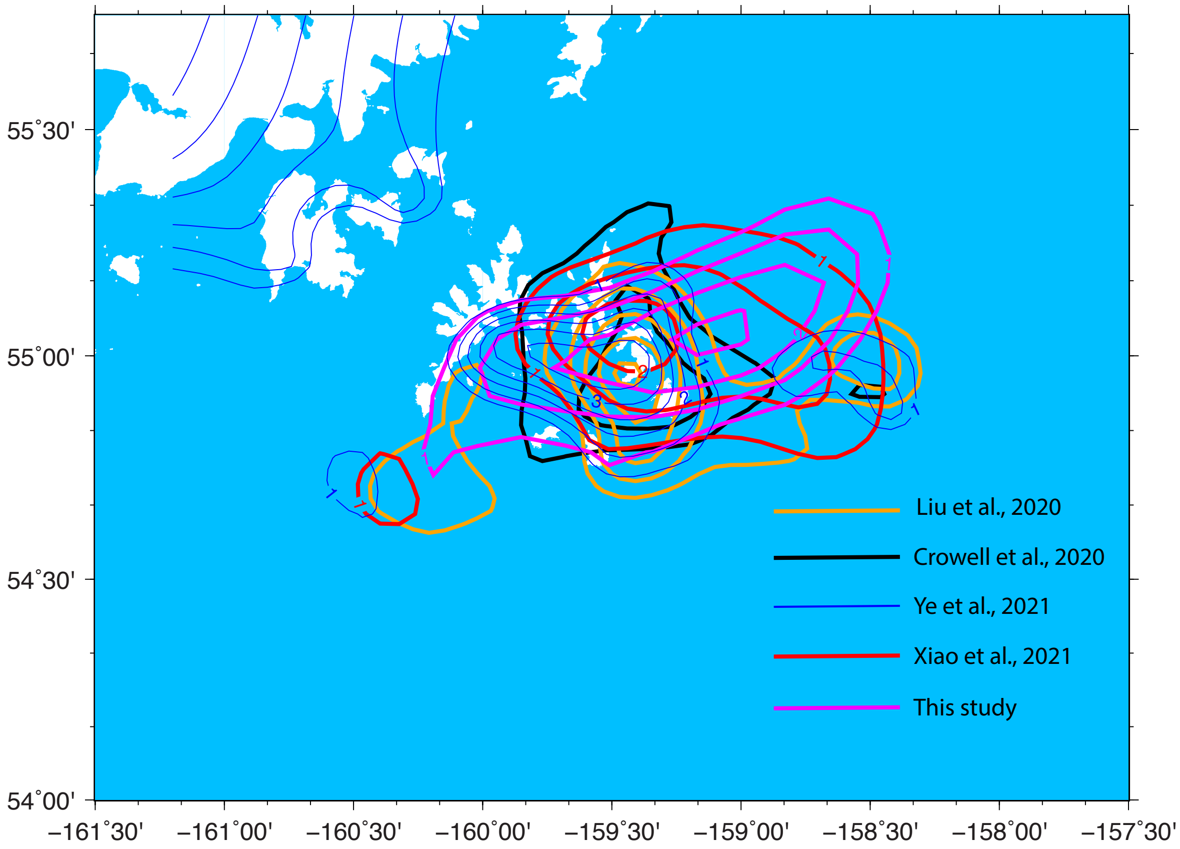

Supplementary Fig. 20. Comparison of our model with different published coseismic slip distributions[27-30] with 0.5-m slip contour intervals.

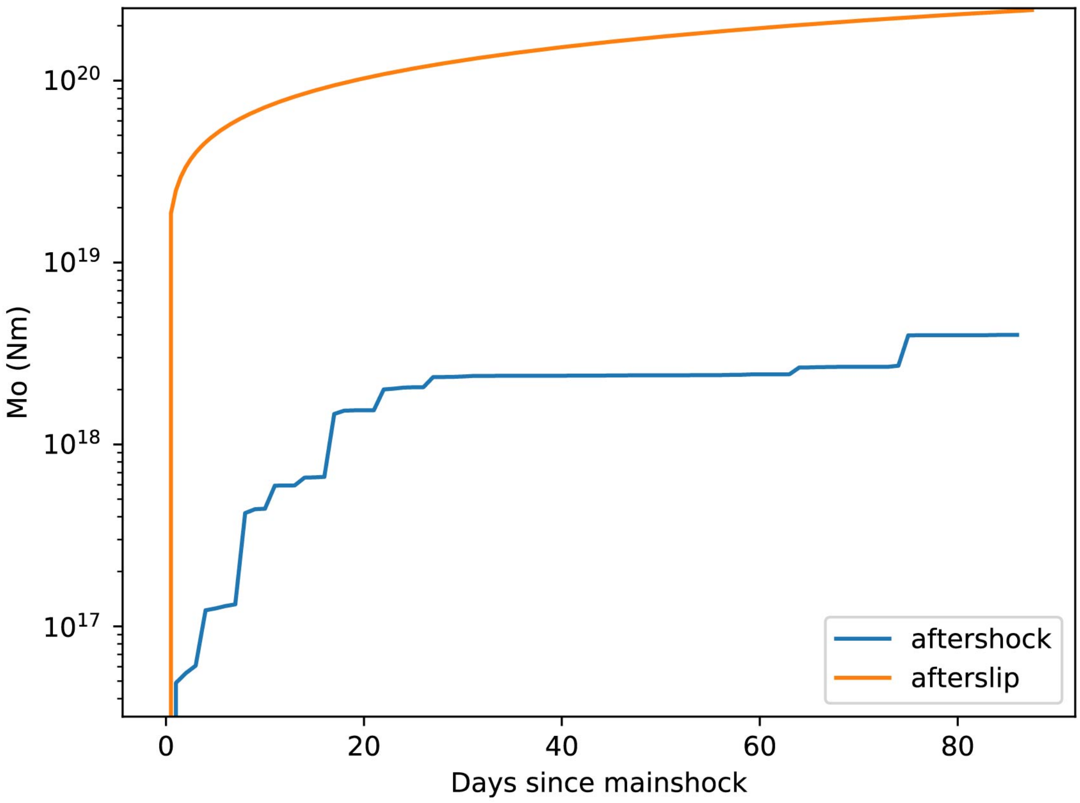

Supplementary Fig. 21. Cumulative seismic moment (M_0) released by aftershocks following the 2020 Mw 7.8 Simeonof earthquake compared to time-dependent aseismic afterslip moment determined in the third scenario of the frictional afterslip model (afterslip fully surrounding the rupture).

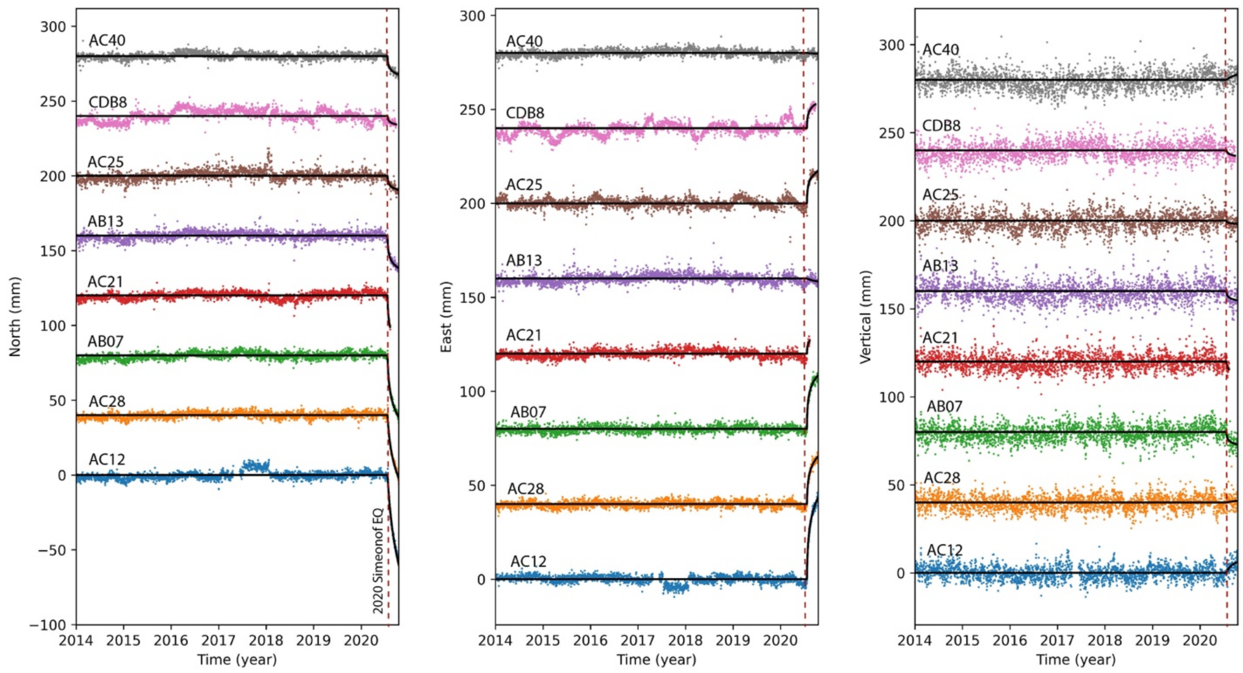

Supplementary Fig. 22. Position time series of selected GPS sites with absolute magnitude of coseismic offsets larger than 2.0 cm. The estimated long-term velocities, seasonal variations, and coseismic offsets (first seven terms in Equation 1) are removed from the raw data (dots) and from predicted time series using equation (1) in the main text. Dashed vertical red lines represent the occurrence of the 2020 Mw 7.8 Simeonof earthquake.

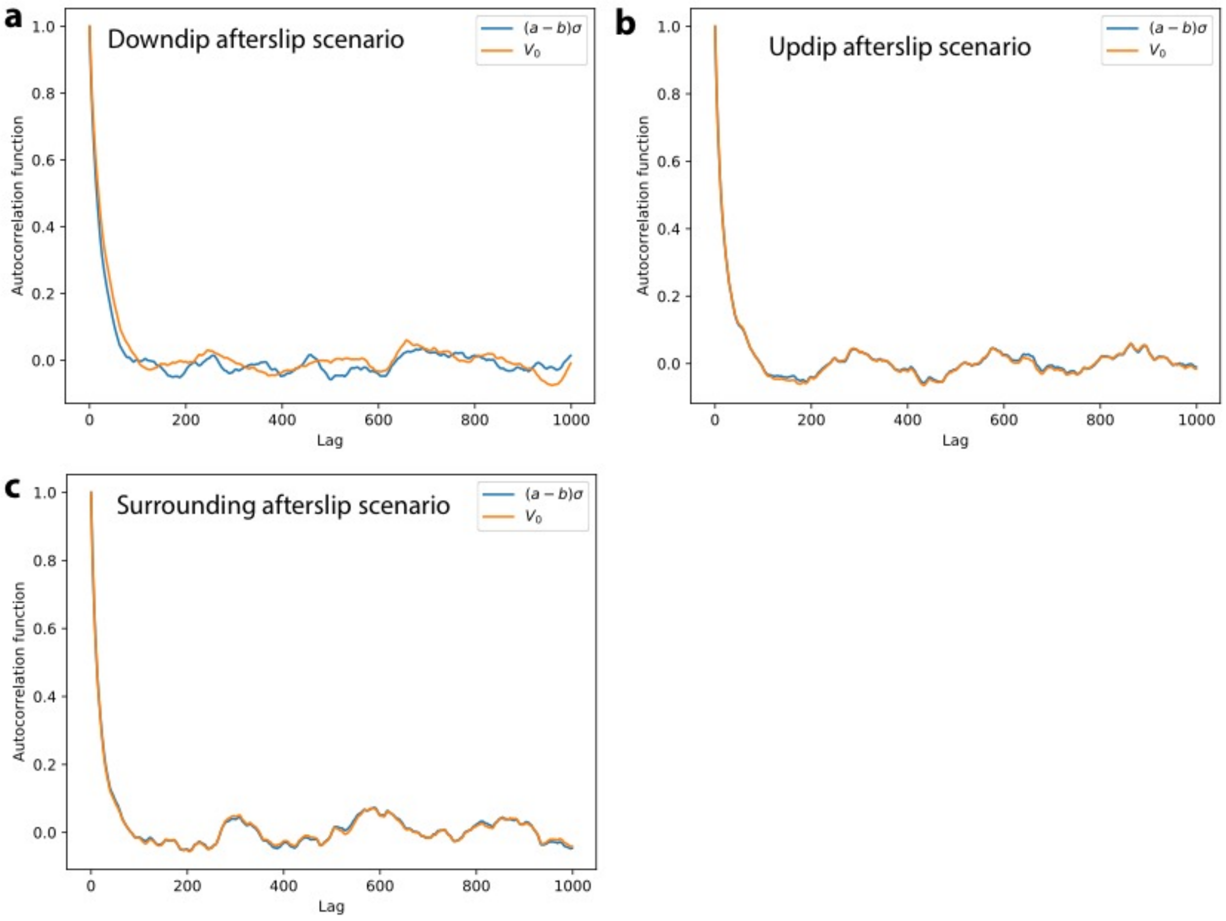

Supplementary Fig. 23. The normalized autocorrelation function of the stochastic process that generated the chains for estimating the frictional parameter and reference velocity. Results for three scenarios of downdip-only, updip-only and fully surrounding the coseismic slip zone are shown in **a**, **b** and **c**, respectively.
